# Air pollution exposure and its effects on idiopathic pulmonary fibrosis: clinical worsening, lung function decline, and radiological deterioration

**DOI:** 10.3389/fpubh.2023.1331134

**Published:** 2024-01-10

**Authors:** Pablo Mariscal-Aguilar, Luis Gómez-Carrera, Gema Bonilla, Mariana Díaz-Almirón, Francisco Gayá, Carlos Carpio, Ester Zamarrón, María Fernández-Velilla, Isabel Torres, Isabel Esteban, Rita Regojo, Elena Villamañán, Concepción Prados, Rodolfo Álvarez-Sala

**Affiliations:** ^1^Department of Respiratory Medicine, Hospital Universitario La Paz, Madrid, Spain; ^2^Instituto de investigación del Hospital Universitario La Paz (IdiPAZ), Madrid, Spain; ^3^Universidad Autónoma de Madrid, Department of Medicine, Madrid, Spain; ^4^Department of Rheumatology, Hospital Universitario La Paz, Madrid, Spain; ^5^Department of Radiology, Hospital Universitario La Paz, Madrid, Spain; ^6^Department of Pathology, Hospital Universitario La Paz, Madrid, Spain; ^7^Department of Pharmacy, Hospital Universitario La Paz, Madrid, Spain

**Keywords:** air pollution, idiopathic pulmonary fibrosis, clinical worsening, lung function decline, radiological deterioration

## Abstract

**Introduction:**

Major urban pollutants have a considerable influence on the natural history of lung disease. However, this effect is not well known in idiopathic pulmonary fibrosis (IPF).

**Aim:**

This study aimed to investigate the effects of air pollution on clinical worsening, lung function, and radiological deterioration in patients with IPF.

**Methods:**

This exploratory retrospective cohort study included 69 patients with IPF, monitored from 2011 to 2020. Data on air pollution levels, including carbon monoxide (CO), nitrogen dioxide (NO_2_), particulate matter ≤ 2.5 μM (PM_2.5_), ozone (O_3_), and nitrogen oxides (NO_x_), were collected from the nearest air quality monitoring stations (<3.5 km from the patients' homes). Patient outcomes such as clinical worsening, lung function decline, and radiological deterioration were assessed over various exposure periods (1, 3, 6, 12, and 36 months). The statistical analyses were adjusted for various factors, including age, sex, smoking status, and treatment.

**Results:**

There was an association between higher O_3_ levels and an increased likelihood of clinical worsening over 6 and 36 months of exposure (odds ratio [OR] and 95% confidence interval [CI] = 1.16 [1.01–1.33] and OR and 95% CI = 1.80 [1.07–3.01], respectively). Increased CO levels were linked to lung function decline over 12-month exposure periods (OR and 95% CI 1.63 = [1.01–2.63]). Lastly, radiological deterioration was significantly associated with higher CO, NO_2_, and NO_x_ levels over 6-month exposure periods (OR and 95% CI = 2.14 [1.33–3.44], OR and 95% CI = 1.76 [1.15–2.66] and OR and 95% CI = 1.16 [1.03–1.3], respectively).

**Conclusion:**

This study suggests that air pollution, specifically O_3_, CO, NO_2_, and NO_x_, could affect clinical worsening, lung function, and radiological outcomes in patients with IPF. These findings highlight the potential role of air pollution in the progression of IPF, emphasizing the need for further research and air quality control measures to mitigate its effects on respiratory health.

## 1 Introduction

Idiopathic pulmonary fibrosis (IPF) is the predominant progressive fibrotic lung disease within the spectrum of idiopathic interstitial pneumonias (1–3). This disease is characterized by the replacement of healthy tissue with altered extracellular matrix, leading to the destruction of alveolar architecture, decreased lung compliance, and ultimately, respiratory failure and death. Patients diagnosed with IPF have a median survival of approximately 3–5 years from the time of diagnosis. A number of mortality predictors have been found, including older age and smoking status, and research has recently suggested that air pollution is a significant prognostic factor in the natural history of IPF (4–10).

Air pollution is the presence of physical, chemical, or biological agents in concentrations that change the atmosphere's structure, which can have a multitude of short- and long-term consequences on human health. Air pollution is a leading cause of premature death, accounting for approximately 300,000 deaths annually in Europe (11), with the primary causes including cardiovascular and respiratory diseases. Urban air pollution has been associated with a higher incidence of chronic obstructive pulmonary disease (COPD) and asthma exacerbations, reduced lung function in lung diseases such as COPD, and increased mortality of patients with respiratory disease (12–15).

Activities such as intensive agriculture, deforestation, industrial processes, and the increasing use of motor vehicles expose the respiratory system to increased concentrations of polluted air (16). Certain pollutants have been demonstrated to cause lung deterioration, including carbon monoxide (CO), nitrogen dioxide (NO_2_), particulate matter (PM_2.5_), ozone (O_3_), and nitrogen oxides (NO_x_), which are among the most important pollutants in terms of their effects on the respiratory system (11).

Certain lung defense mechanisms, including mucociliary clearance and macrophage function, can be affected by polluted air, whose particles lead to direct irritation and inflammation throughout the entire respiratory system, from the nasal passages down to the alveolar sacs, and elevate oxidative stress through DNA modifications (17). Some protocols have studied the relationship between air pollution and IPF in terms of exacerbations, mortality, and lung function (10, 18–20).

We postulated that major urban pollutants could worsen the symptoms, lung function, and radiological pattern of patients with IPF; however, we found no other studies that had examined the association between air pollution and the clinical worsening and radiological deterioration of IPF. Additionally, the exposure period necessary to develop these effects in patients with IPF differs among studies, and no specific time frame has been established. Just as Mariscal-Aguilar et al. (10), we examined pollution levels over periods of 1, 3, 6, 12, and 36 months prior to an event, given that assessing average air pollution levels over extended periods has frequently been shown to yield more robust connections with respiratory alterations. The decision to publish two different papers was because each article addresses a different objective: the initial publication primarily focused on acute alterations observed during patient follow-up, emphasizing immediate impacts, such as exacerbations and hospitalizations. In contrast, the current article delves into long-term alterations in outpatient follow-up, examining outcomes over extended periods. This division of papers allows for a more nuanced exploration of the varying impacts of air pollution on the trajectory of IPF, ensuring a more comprehensive understanding of the temporal dynamics and multifaceted nature of environmental influences on IPF progression.

## 2 Materials and methods

### 2.1 Study design and participants

We conducted an exploratory retrospective cohort study of 69 patients followed up in the Interstitial Lung Disease Unit of La Paz University Hospital from 2011 to 2020. The inclusion criteria were a diagnosis of IPF according to the guidelines of the American Thoracic Society (ATS)/European Respiratory Society (ERS)/Japan Radiological Society/Latin American Thoracic Association (1, 2) and an age between 18 and 90 years. The exclusion criteria were a lack of affiliation or medical data, a change of residence, and an absence from more than 2 clinical visits (Table 1). Medical assessments were performed every 3–4 months according to clinical practice guidelines. Clinical data were obtained from the patients' medical records, and their dyspnea levels, lung function tests, radiological images, other diagnoses, and antifibrotic therapy were recorded. Patients were considered to be undergoing antifibrotic therapy if the treatment lasted more than 45 days (10, 21).

**Table 1:** Inclusion and exclusion criteria.

**Inclusion criteria**	**Exclusion criteria**
Diagnosis of IPF according to ATS/ERS/JRS/LAT guidelines	Lack of affiliation or medical data
Age between 18 and 90 years	Change of residence
Underwent regular medical assessments every 3–4 months	Absence from more than 2 clinical visits
Dyspnea levels, lung function tests, radiological images, other diagnoses, antifibrotic therapy	

### 2.2 Air pollution data

The Government of Madrid and the Community of Madrid's Integral Air Quality System performs hourly measurements of each pollutant through air quality monitoring stations. The pollutants NO_2_, PM_2.5_, O_3_, and NO_x_ were measured in μg/m^3^, whereas CO was measured in mg/m^3^ (22, 23). The monitoring station nearest to each patient's home was selected for collecting air quality data. If a specific pollutant was not monitored at a station, patients were linked to an alternative station nearby, provided it was within 3.5 km of their residence. Google Maps' geographical coordinates were employed to calculate the distance between the monitoring stations and each patient's home (10), using the inverse of the square of the distance for the analysis.

### 2.3 Outcome assessment

Clinical deterioration was defined as an increase in the modified Medical Research Council (mMRC) dyspnea scale or the onset of a new cough. Lung function decline was defined as a ≥10% decrease in forced vital capacity (FVC) of the predicted value compared with the previous visit or a ≥15% decrease in diffusing lung capacity for carbon monoxide (DLCO) of the predicted value compared with the previous visit. These tests, conducted during each consultation (spirometry, carbon monoxide diffusion capacity), were performed in a laboratory that adheres to the ATS/ERS standardized criteria (24, 25). For this purpose, we employed an integrated module in the MasterLab-body version 6.0 equipment (Viasys, Wuerzburg, Germany). The 6-min walk test was performed according to the ATS/ERS recommendations (26).

Radiological worsening was defined by the onset of new ground glass opacities or increasing signs of fibrosis (loss of volume, traction bronchiectasis, or more honeycombing compared with previous high-resolution computed tomography [HRCT] images). HRCT for both diagnosis and monitoring was performed with a 16-detector computed tomography scanner (Somatom Emotion 16; Siemens Medical Solutions, Erlangen, Germany), with all scans conducted by the same radiology staff. All requested echocardiograms were examined by the same members of the Cardiology Service with Philips IE33 and Philips EPIQ echocardiography machines.

### 2.4 Ethics

The study was conducted in accordance with the good clinical practice standards and the ethical principles of the Declaration of Helsinki and was approved by the La Paz University Hospital Clinical Research Ethics Committee (code PI-3742, 20 June 2019). Given the retrospective nature of the study, informed consent was not required.

### 2.5 Statistical analysis

To determine the value of each pollutant in relation to the interval between patient visits, the mean level of each pollutant was weighted by the inverse of the square of the distance between the patient's residence and the nearest air quality station, up to a maximum distance of 3.5 km.

Correlated data were examined with a generalized linear mixed model employing the restricted maximum pseudo-likelihood approach. Three different endpoints were evaluated: clinical worsening, lung function test, and radiological progression. To estimate the “likelihood of the event,” a random intercept and an unstructured covariance matrix were incorporated into the generalized linear mixed model, using a binomial distribution and a logit link function. Each air pollutant was introduced into the model to assess its association with the binary outcome in terms of odds ratios (ORs). To estimate the mean air pollutant levels, another random intercept and unstructured covariance matrix were included in the generalized linear mixed model, employing a normal distribution and an identity link function.

The statistical models used were adjusted for age, sex, smoking status, FVC, the initial visit's DLCO, the antifibrotic therapy regimen, and the presence of pulmonary hypertension.

In general, 2-tailed *p*-values <0.05 were deemed statistically significant. The analyses were performed with R Statistical Software V4.0.4 (2021-02-15) with the mgcv package (mgcv_1.8-33) and SAS Enterprise Guide 8.2 statistical software from SAS Institute Inc. (Cary, NC, USA).

## 3 Results

Of the initial 71 patients enrolled in the cohort, 2 were excluded because they resided more than 3.5 km from the nearest station. The included patients' baseline characteristics are listed in Table 2. The patients' mean age was 73.7 ± 7.7 years, 53 (76.8%) of them were men, and 39 (56.5%) were never smokers. On the other hand, 50 (72.4%) patients experienced clinical worsening during the follow-up, 55 (79.7%) had a decline in lung function, and 30 (43.5) experienced radiological worsening.

**Table 2:** Patients' baseline characteristics.

**Baseline characteristics**	**Mean ±standard deviation or number (percentage)**
Age, years	73.70 ± 7.72
**Sex, number (%)**	
Women, number (%)	16 (23.2)
Men, number (%)	53 (76.8)
Height, cm	72.85 ± 14.12
Weight, kg	165.5 ± 10.32
Body mass index, kg/m^2^	26.40 ± 3.92
**Smokers, number (%)**	
Non-smoker	39 (56.5)
Current smoker	30 (43.5)
Packs per year	22.65 ± 13.97
Charlson Index	2 ± 1.5
Dyspnea (modified Medical Research Council)	1 ± 0.65
Forced expiratory volume in the first second (FEV_1_) % prebronchodilator, % predicted	88.20 ± 18.3
Forced vital capacity (FVC) % prebronchodilator, % predicted	82.42 ± 18.39
Diffusing capacity of the lungs for carbon monoxide (DLCO), % predicted	63.45 ± 20.54
Carbon monoxide transfer coefficient (KCO), % predicted	91.8 ± 17.9
6-min walk test, m	488.89 ± 101.69
Initial oxygen saturation, %	94 ± 2.2
Final oxygen saturation, %	85.83 ± 5.2
pH	7.42 ± 0.01
Partial pressure of oxygen in baseline arterial blood, mm Hg	72.40 ± 10
Partial pressure of carbon dioxide in baseline arterial blood, mm Hg	37.4 ± 4.1
Systolic pulmonary artery pressure (sPAP)	32.6 ± 7.16
Antifibrotic therapy >45 days	61 (88.4)
Pirfenidone >45 days	48 (69.5)
Nintedanib >45 days	21 (30.5)
Patients with at least 1 episode of clinical worsening	50 (72.4)
Patients with at least a decrease in lung function (FVC or DLCO)	55 (79.7)
Patients with at least 1 episode of radiological deterioration	30 (43.5)
Chronic respiratory failure	22 (31.9)
Hospitalizations (respiratory cause)	29 (42)
Deaths	22 (31.9)

### 3.1 Effects of air pollution on clinical worsening in patients with idiopathic pulmonary fibrosis

**Figure 1:**
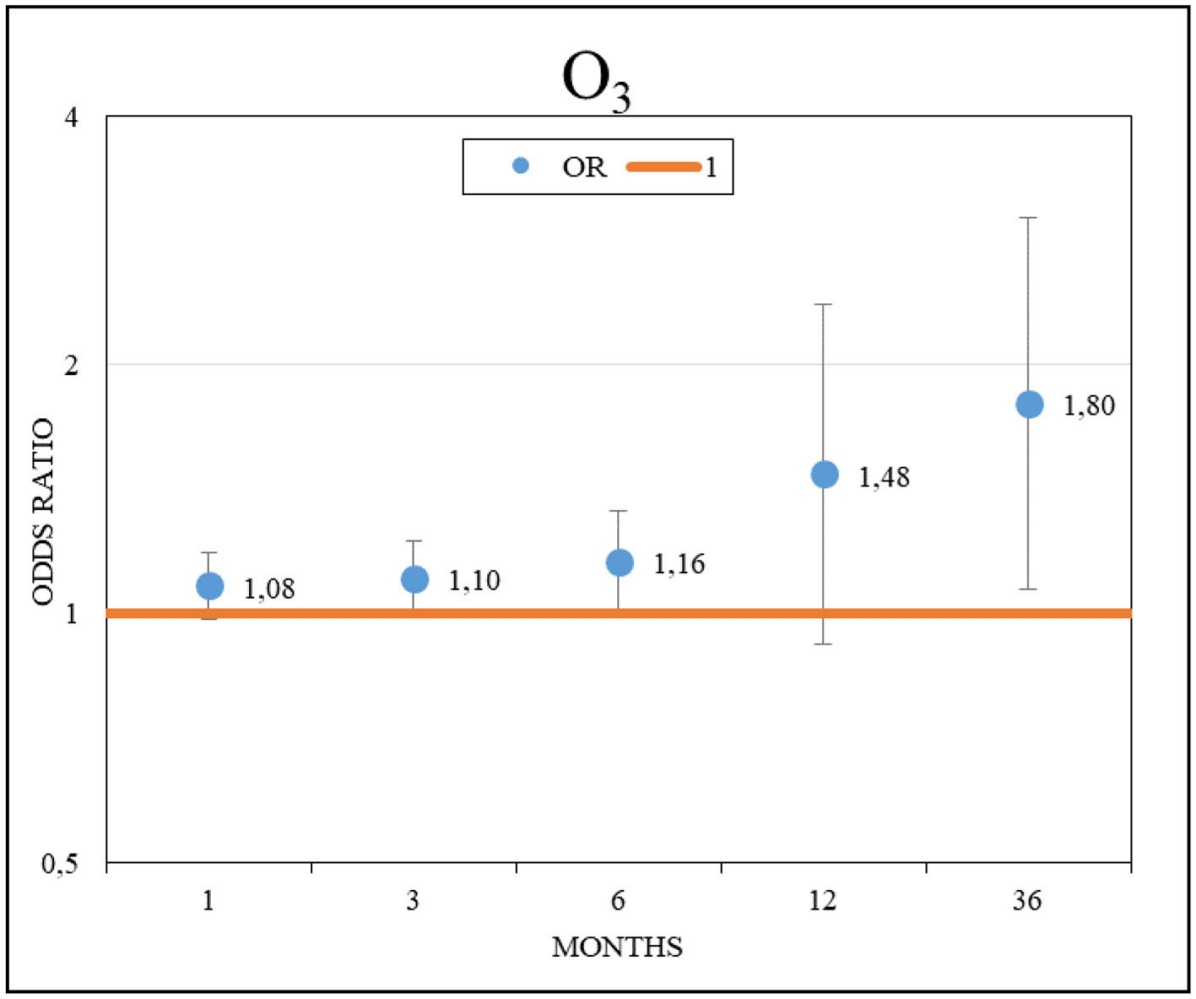
Effects of O_3_ on clinical worsening in patients with idiopathic pulmonary fibrosis.

The increase in the cumulative mean O_3_ values was significantly associated with an increased likelihood of worsening symptoms. An odds ratio (OR) and 95% confidence interval (CI) = 1.16 [1.01–1.33] (*p* = 0.04) was observed for each 10 μg/m^3^ increment during the cumulative exposure of the previous 6 months, and an OR and 95% CI = 1.80 [1.07–3.01] (*p* = 0.02) was observed for the 36 months leading up to exacerbation (Figure 1). Conversely, no significant association was observed between the likelihood of clinical exacerbation and the other pollutants studied.

### 3.2 Effects of air pollution on lung function in patients with idiopathic pulmonary fibrosis

**Figure 2:**
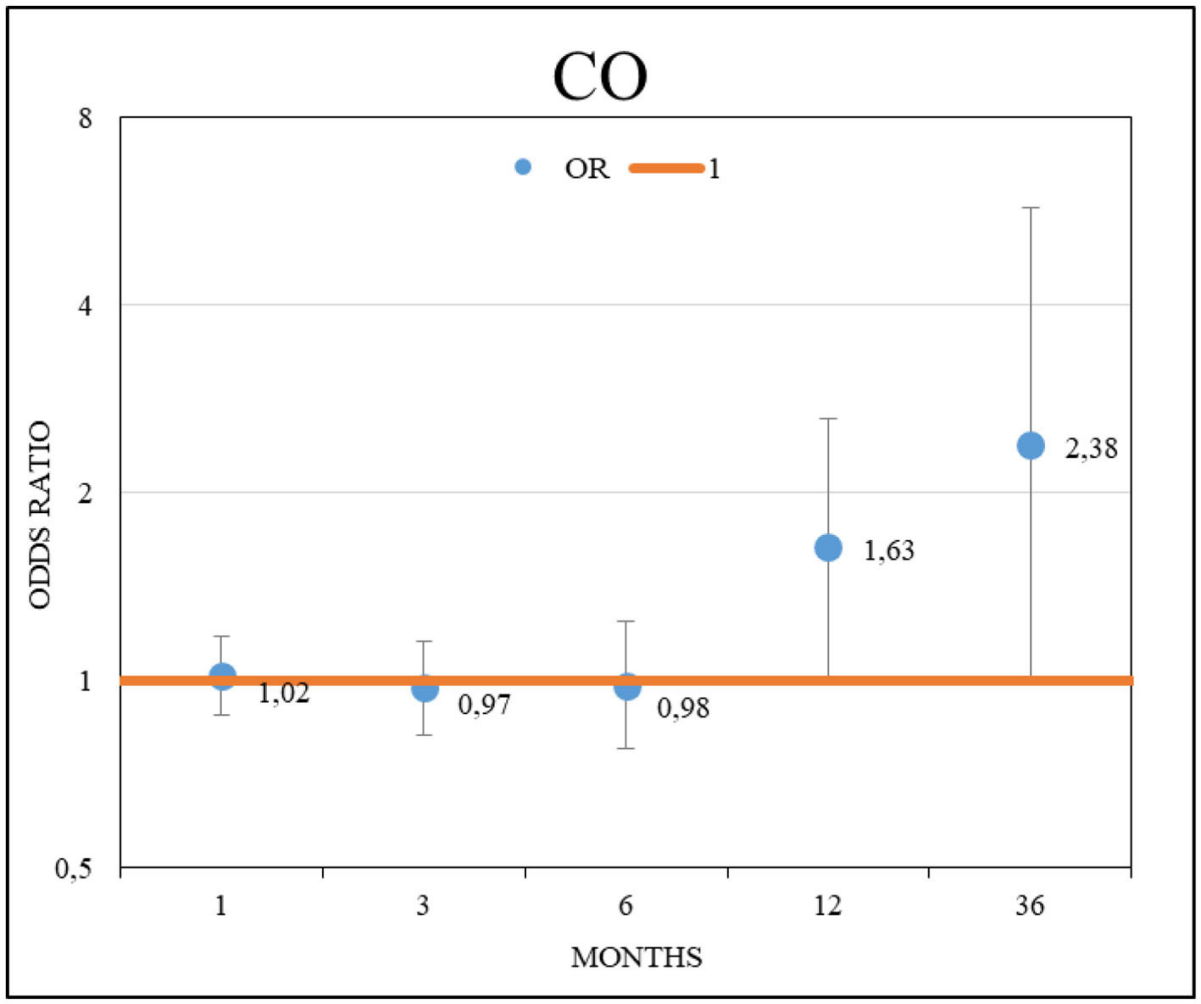
Effects of CO on lung function decline on patients with idiopathic pulmonary fibrosis.

The increase in the cumulative mean CO values was significantly associated with an increased likelihood of worsening lung function. An odds ratio (OR) and 95% confidence interval (CI) = 1.63 [1.01–2.63] (*p* = 0.04) was observed for each 0.1 mg/m^3^ increment during a 12-month cumulative exposure leading up to this deterioration (Figure 2). However, no association was detected between the likelihood of worsening lung function and the other evaluated pollutants.

### 3.3 Effects of air pollution on radiological deterioration in patients with idiopathic pulmonary fibrosis

**Figure 3:**
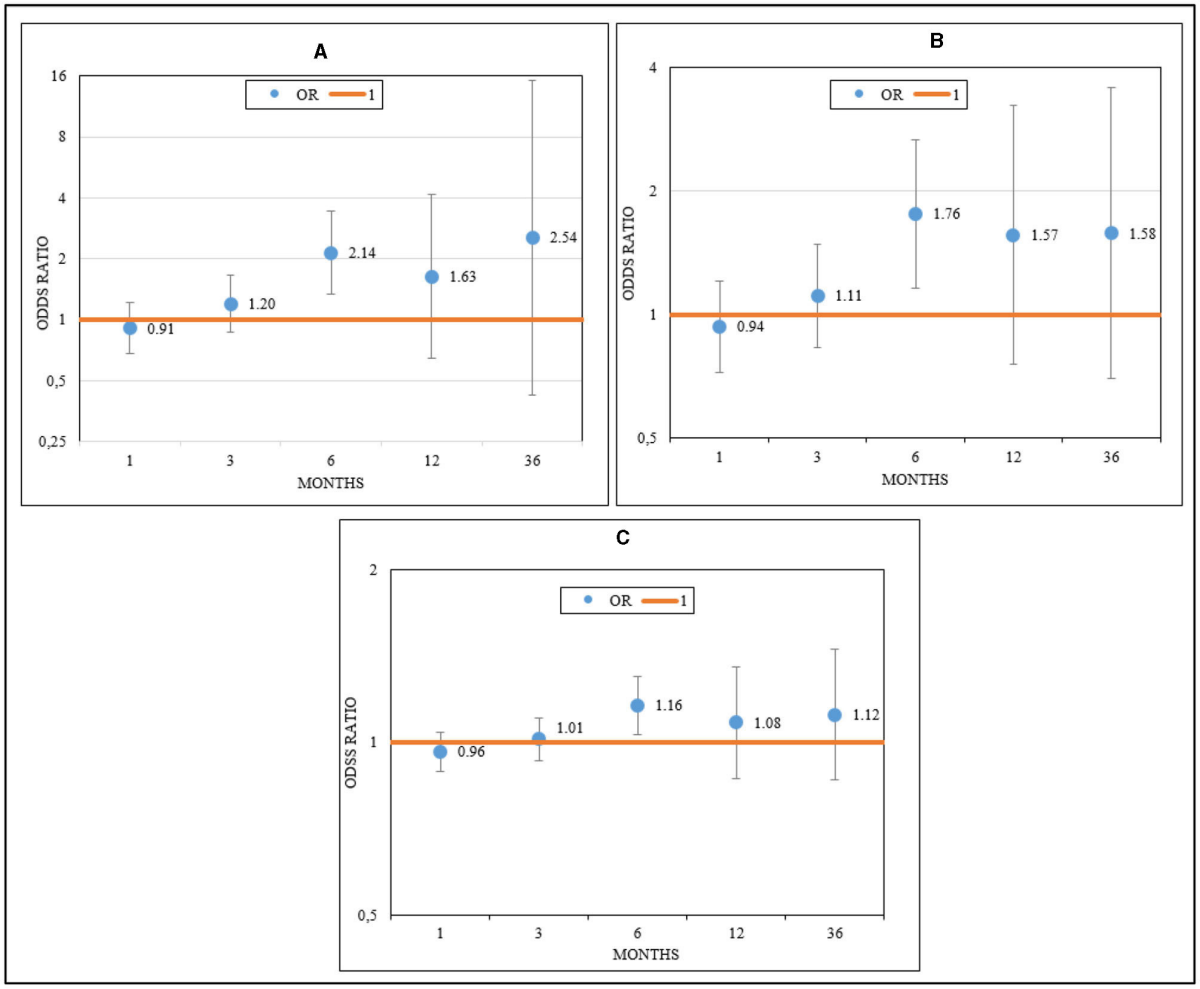
Effects of CO **(A)**, NO_2_ **(B)**, and NO_x_ **(C)** on radiological deterioration in patients with idiopathic pulmonary fibrosis.

The increase in cumulative mean CO, NO_2_, and NO_x_ values was significantly associated with an increased probability of radiological worsening. An odds ratio (OR) and 95% confidence interval (CI) = 2.14 [1.33–3.44] (*p* < 0.01) was observed for each 0.1 mg/m^3^ increment in the case of CO; for NO_2_, an OR and 95% CI = 1.76 [1.15–2.66] (*p* < 0.01) was observed for each 10 μg/m^3^ increment; and for NO_x_, an OR and 95% CI = 1.16 [1.03–1.3] (*p* = 0.01) was noted, all with a cumulative exposure of 6 months leading up to deterioration (Figures 3A–C).

## 4 Discussion

Our results indicate that, regardless of other factors such as age, sex, smoking habits, lung function, and antifibrotic therapy, there could be a relationship between air pollution and clinical worsening in patients with IPF. Although the observed proportion of smokers (43.5%) among the patients enrolled in our study might appear comparatively lower in contrast to some studies reporting a stronger association between smoking and IPF (27, 28), this discrepancy could be attributed to the multifaceted nature of IPF pathogenesis. Smoking indeed plays a pivotal role as a risk factor; however, it is not the exclusive determinant in all cases. Genetic predisposition, environmental exposures beyond smoking, occupational hazards, and geographic variations significantly contribute to the heterogeneous nature of IPF etiology. Our study's selection criteria focused specifically on patients diagnosed with IPF, which might have led to a particular subset of individuals participating in the research, possibly differing from broader population samples.

On the other hand, the findings suggest that higher mean O_3_ levels are associated with an increased likelihood of clinical deterioration during various exposure periods (6 and 36 months preceding the event). To date, no studies in the reviewed literature have investigated the link between O_3_ pollution and the likelihood of clinical deterioration in patients with IPF. There is, however, a study that analyzed air pollution and dyspnea level in these types of patients but found no statistically significant association. Johannson et al. (8) employed the University of California San Diego Shortness of Breath Questionnaire (UCSD-SOBQ) and found no relationship between mean air pollutant levels and changes in this questionnaire. The authors' results differed from ours because the outcome was measured with different questionnaires that were not comparable with ours. In addition, the UCSD-SOBQ questionnaire includes different items, and these cover only dyspnea in various situations. In addition to the mMRC dyspnea scale, our study included cough as an important symptom that negatively affects the quality of life of patients with IPF.

As far as lung function is concerned, our cohort's patients with decreased respiratory function were exposed to higher CO concentrations, such that for every 0.1 mg/m^3^ increase in CO, the probability of functional deterioration was 1.63-fold higher, with an exposure period of 12 months. Ours is the first study to measure and confirm the influence of CO on changes in respiratory function in patients with IPF.

A number of studies have analyzed the relationship between air pollution and the likelihood of declining lung function in IPF, such as Winterbottom et al. (7), who demonstrated a significant relationship in PM_10_: for every 1 μg/m^3^ increase, there was a decrease of 46 cubic centimeters (cc) per year in FVC, with no positive results related to PM_2.5_. In contrast, Zheng et al. (29) confirmed a significant effect for elevated PM_2.5_ levels with long-term exposure and a decrease in DLCO; however, they did not observe any correspondence with other air pollutants. Yoon et al. (30) demonstrated an association between air pollution (increased NO_2_ levels) and decreased FVC, whereas Johannson et al. (8) and Sesé et al. (4) found no relationship between air pollution (NO_2_, PM_2.5_, PM_10_, and O_3_) and decreased lung function.

Although Winterbottom et al. (7) found no relationship with PM_2.5_, they found one with PM_10_. Our study could not include PM_10_ data due to insufficient density of air pollution stations in Madrid. The results of Zheng et al. (29) partially differ from ours because the data are positive only for the DLCO test and long-term exposure (5 years); for FVC, the authors did not observe any relationship. Therefore, more information is needed, which can be obtained by installing more monitoring stations or by increasing the distance between the patients' homes and the measurement stations, which in turn could reduce the accuracy of the other pollutants. Similar studies have shown consistent results: similar to our protocol, Johannson et al. (8) and Sesé et al. (4) found no significant association between the levels of various analyzed pollutants (NO_2_, PM_2.5_, PM_10_, and O_3_) and the decline in lung function. However, Yoon et al. (30) found a significant relationship between NO_2_ levels and the decline in FVC, results that differ from our protocol's results for 2 main reasons: First, we used the cumulative mean, whereas Yoon et al. estimated the 2-year annual concentrations and employed prediction models for the following 15 years. Second, our study had a substantially smaller sample size.

One of the aspects to consider in monitoring IPF progression is the radiological change these patients undergo over time. We found no studies that exclusively analyzed the deterioration of patients with IPF at the radiological level in relation to atmospheric pollution levels. Our results reveal a significant relationship between the increase in cumulative mean levels of CO, NO_2_, and NO_x_ and the deterioration observed in the radiological images over a 6-month exposure period.

Despite the lack of similar studies with which to make direct comparisons and to contrast with our data, there was 1 study that examined the occurrence of subclinical interstitial lung disease, defined by pollution-related interstitial lung abnormalities. Sack et al. (31) demonstrated an increased incidence of interstitial lung abnormalities in healthy individuals, which was associated with prolonged exposure to NO_2_ and NO_x_ over 10–30 years. However, these results should be interpreted with caution. Although we are assessing the radiological progression of an already established and diagnosed disease, Sack et al. (31) examined isolated radiological features that are insufficient for considering the image as part of a specific pulmonary condition. Furthermore, the exposure time, and therefore the methods employed, differed; in our case, we analyzed up to 36 months of exposure, whereas Sack et al. (31) employed predictive models spanning up to 30 years.

The varied intervals at which the ORs between air pollutants and clinical worsening, lung function decline, and radiological deterioration changes over several exposure periods are likely due to multifaceted interactions between environmental factors and the intricate disease course of IPF. This nuanced relationship could be attributed to several key aspects. First, the differential impact observed in clinical worsening over 6 and 36 months, as well as in lung function decline over 12 months, could reflect the temporal nature of disease progression and the duration of exposure required to manifest significant alterations. The initial impact of pollutants on clinical symptoms and lung function might be more acute within shorter intervals, during which the effects might be more immediately noticeable. Conversely, longer-term exposure periods might reflect a cumulative effect, leading to adaptations in disease processes or patient responses, potentially diminishing the observed associations. Second, the disparity in radiological worsening after 6 months might be explained by the nature of radiological changes in IPF. Early responses to pollutants might trigger observable changes; however, as the disease progresses or stabilizes, the immediate influence of pollutants on these radiological manifestations might attenuate, potentially explaining the reduction in ORs after 6 months. Additionally, the evolving disease trajectory, potential treatment responses during the follow-up period, and individual variability in patient responses to pollutants and disease progression could contribute to the fluctuating ORs across varying exposure durations. These complexities underscore the intricate nature of the relationship between air pollutants and various aspects of IPF progression, in which the action of various biological pathways, oxidative stress, inflammation, and other interactions with the respiratory system are important. Thus, a comprehensive understanding of temporal dynamics and disease interactions is necessary to interpret the observed variations in ORs across distinct exposure periods.

On the other hand, comparing these findings with the investigation by Mariscal-Aguilar et al. (10) is crucial. The substantial findings from both studies have significant implications for public health interventions and managing patients with IPF. These investigations underscore a need for a paradigm shift in patient care strategies, particularly understanding how air pollution influences clinical worsening, lung function decline, and radiological deterioration in IPF patients. It's also essential to recognize the correlation between air pollution and increased hospitalizations or mortality in IPF, which further emphasizes the need for healthcare providers to integrate environmental exposure assessments into routine patient evaluations. This integration should include personalized counseling to reduce exposure to high levels of air pollutants, part of patient education programs. Additionally, these findings highlight the importance of early detection and prompt intervention in managing IPF patients exposed to environmental pollutants. Clinicians should be vigilant for exacerbations or lung function declines in patients in polluted areas and adjust management strategies, possibly considering more aggressive therapeutic interventions or closer monitoring. Such measures could potentially reduce IPF patient mortality.

There are a number of limitations to this study. The sample size was small due to its single-center nature; however, it is important to consider the low prevalence of IPF. Also, we could not include humidity and temperature information because it was not available for all areas during the study period. The most common limitation in this type of study, similar to other air pollution protocols, is the lack of exposure measurements in the work area. Lastly, despite having analyzed the most important air pollutant to the respiratory system, it was not feasible to control variables such as other particles that could have had an effect on the progression of patients with IPF.

Nevertheless, this protocol has a number of strengths. First, the outcomes considered in the study (clinical worsening, lung function, radiological deterioration) are critical for performing proper clinical follow-up of these patients. Second, if we compare our study with the literature related to air pollution's effects on IPF, our study examined the largest variety of particles (CO, NO_2_, PM_2.5_, O_3_, and NO_x_). Third, our research provides the highest level of precision for the measurements regarding the patients' residence, given that a maximum distance of 3.5 km was established between the residence and the air quality station. Fourth, various exposure periods were considered to define how much time would be necessary for exposure to any air pollutant to change the clinical course of these patients.

## 5 Conclusion

The results of this study indicate that environmental pollution, particularly stemming from 5 major pollutants (CO, NO_2_, O_3_, and NO_x_), could significantly affect clinical worsening, lung function decline, and radiological deterioration in patients with IPF. These findings align with the World Health Organization's recommendations to reduce pollutant emissions and could stimulate changes in environmental policies to enhance the monitoring and clinical progression of these patients. It is essential to continue this line of research with future protocols that enable more accurate quantifications of ambient air quality, with a particular focus on these pollutants.

## Data availability statement

The original contributions presented in the study are included in the article/supplementary material, further inquiries can be directed to the corresponding author.

## Author contributions

PM-A: Conceptualization, Data curation, Formal analysis, Methodology, Resources, Software, Validation, Visualization, Writing—original draft, Writing—review & editing, Funding acquisition. LG-C: Conceptualization, Investigation, Supervision, Writing—original draft, Writing—review & editing. GB: Data curation, Writing—original draft, Writing—review & editing. MD-A: Formal analysis, Resources, Software, Writing—review & editing, Methodology. FG: Formal analysis, Resources, Software, Writing—review & editing, Methodology. CC: Validation, Writing—review & editing, Formal analysis, Resources, Software, Supervision. EZ: Investigation, Visualization, Writing—original draft. MF-V: Writing—review & editing, Validation. IT: Writing—review & editing, Validation. IE: Visualization, Writing—review & editing. RR: Writing—review & editing, Visualization. EV: Investigation, Writing—review & editing. CP: Conceptualization, Visualization, Writing—review & editing. RÁ-S: Conceptualization, Funding acquisition, Investigation, Methodology, Project administration, Resources, Supervision, Validation, Visualization, Writing—original draft, Writing—review & editing.
